# Effects of dietary amylose/amylopectin ratio on antioxidant ability and amino metabolism in the liver of weaned piglets undergoing feed transition and challenged with lipopolysaccharide

**DOI:** 10.3389/fnut.2024.1435051

**Published:** 2025-01-06

**Authors:** Can Yang, Xiaowu Tang, Min Wang, Han Yang, Huansheng Yang, Yancan Wang, Yulong Yin

**Affiliations:** ^1^College of Life Sciences, Hunan Provincial Key Laboratory of Biological Resources Protection and Utilization in Nanyue Mountain Area, Hengyang Normal University, Hengyang, Hunan, China; ^2^Hunan International Joint Laboratory of Animal Intestinal Ecology and Health, Hunan Normal University, Changsha, Hunan, China; ^3^College of Bioengineering, Hunan Vocational Technical College of Environment and Biology, Hengyang, Hunan, China; ^4^Key Laboratory of Agro-Ecological Processes in Subtropical Region, Institute of Subtropical Agriculture, Chinese Academy of Science, Changsha, Hunan, China

**Keywords:** dietary amylose/amylopectin ratio, LPS stress, amino acid, antioxidant activity, weaned piglet

## Abstract

To find out whether dietary amylose/ amylopectin ratio (DAR) could attenuate injury in lipopolysaccharide (LPS)-challenged piglets, sixty male weaned piglets (Duroc × Landrace × Yorkshire, 21 days old, 6.51 ± 0.64 kg) were allotted to 5 dietary treatments with 12 cages per treatment, and fed ad libitum with diets different in DAR (0.00, 0.20, 0.40, 0.60 and 0.80). Feed transformation occurred from D15 to D21. On day 28, 12 h before slaughter, pigs were intraperitoneal injected with 100 μg/kg body weight LPS or sterile saline. Results showed that LPS stress caused an increase in serum urea nitrogen (UREA) and triglyceride (TG), but a decrease in alanine aminotransferase (ALT) activity and glucose (GLU) concentration (*p* < 0.05). Serum immunoglobulin G (IgG) concentration increased in DAR 0.80 but decreased in other groups after LPS stress (*p* < 0.05). Compared with the control group, concentrations of Ile, Leu, Phe, Val, Thr, Arg decreased in serum but increased in liver after LPS stress (*p* < 0.05). Serum Arg, Tyr, Sar, Ans, Orn increased linearly with increasing DAR (*p* < 0.05). Piglets in diet DAR 0.00 had highest superoxide dismutase (SOD1) and glutathione peroxidase 1 (*GPX1*) mRNA expression in liver than those in other groups (*p* < 0.05). There was significant effect of LPS stress * dietary DAR on total SOD activity and SOD1 mRNA gene expression (*p* < 0.05), LPS stress caused an increase in those two indices for pigs in groups 0.00 and 0.80. Piglets in diet 0.80 had the highest hepatic Cu, Fe, Mn, Zn concentrations than those in other groups (*p* < 0.05). Cecal indol(e) concentration was higher in diet 0.00 than that in diet 0.80 (p < 0.05). After LPS stress, colonic skatole concentration increased in DAR 0.40, 0.80 but decreased in other groups (*p* < 0.05). In conclusion, adverse effects of the LPS challenge could be reversed by feeding weaned piglets with low or high DAR diet through regulating amino metabolism and antioxidant function.

## 1 Introduction

Lipopolysaccharide (LPS) is a major component of the outer membrane of gram-negative bacteria such as *Escherichia coli*. It triggers a systemic inflammatory process by releasing pro-inflammatory mediators, nitric oxide, and reactive oxygen species (ROS), which are associated with oxidative stress (1). Weaned piglets with weakened mucosal barrier resistance to pathogens are particularly vulnerable to infections by enterotoxigenic *E. coli*. This strain of *E. coli* produces and secretes LPS, leading to intestinal dysfunction (2), oxidative stress (3), and hepatic damage, especially at doses like 80 μg/kg body weight of LPS (4). Pigs treated with LPS exhibited hepatocyte nuclear lysis, fibroblast proliferation, and karyopyknosis (5). Furthermore, LPS-induced immune stimulation decreases plasma flux for amino acids such as lysine (Lys), phenylalanine (Phe), and isoleucine (Ile) (6). However, stress biomarkers of inflammation in LPS-challenged piglets can be partially mitigated through supplementation with a 0.3% amino acid mixture containing arginine (Arg), leucine (Leu), valine (Val), and isoleucine (Ile), and cysteine (Cys) (7). Supplementation with tryptophan (Try) has been shown to ameliorate liver damage by reducing activities of alanine aminotransferase (ALT) and aspartate aminotransferase (AST), maintaining the barrier function of the liver and alleviating hepatic oxidative stress in LPS-challenged piglets (5). Additionally, citrulline (Cit) and arginine deiminase (ADI-PEG20) may exert protective effects against inflammation by reducing CD45+ infiltrates in the liver and lowering plasma levels of pro-inflammatory cytokines in the piglets infused with LPS infusion (8).

Starch is composed of amylose and amylopectin. Waxy maize starches exhibit an A-type X-ray diffraction pattern, whereas high-amylose maize shows a B-type pattern (9) and a lower degree of crystallinity than waxy maize starches (10). Compared with waxy maize starch (DAR 0.07) and non-waxy maize starch (DAR 0.19), feeding finishing pigs a pea starch (DAR 0.28) diet downregulated gluconeogenesis, resulting in less fat and more protein deposition in the liver (11). Compared with normal corn starch, supplementing mice with 10% high-amylose maize enhanced the intestinal absorption of calcium, iron, and magnesium (12). Indigestible starch can be converted into short-chain volatile fatty acids (SCFAs) by intestinal microorganisms in the hindgut (13). Increasing carbohydrate availability by cecal corn starch infusion decreases hindgut aromatic amino acid (AAA) metabolism while increasing systemic AAA availability, thereby promoting hypothalamic neurotransmitter synthesis (14). Additionally, the degree of oxidative stress and inflammation was alleviated in chronic kidney disease (CKD) rats after the consumption of 59% high-amylose maize-resistant starch (HAMRS2) (15).

In commercial pig farms, post-weaning diarrhea usually occurs when weaned piglets are exposed to enterotoxigenic *E. coli* after undergoing the transitional weaning periods, which include changes in feed, environment, and separation from the mother. A dose of 100 μg/kg of LPS secreted by *E. coli* has been shown to cause both hepatic damage and gut injury (4). However, whether different dietary amylose/amylopectin ratios (DAR), achieved by blending high-amylose maize starch and waxy maize starch, can differently improve piglets’ resilience under such challenges by regulating amino acid metabolism and liver antioxidant function remains unclear. Thus, this study examined the effect of dietary amylose/amylopectin ratio on the amino acid concentration in serum and liver, liver mineral element content, and liver antioxidant function in weaned piglets under LPS stress. We hypothesized that both high and low DAR diets could improve piglets’ health by modulating amino acid metabolism and antioxidant function during stress.

## 2 Materials and methods

The experimental procedure in this study was reviewed and approved by the Animal Care and Use Committee of Hunan Normal University (ISA-2017-058).

### 2.1 Animals and diets

A total of 60 castrated male pigs (Landrace × Yorkshire, initial average body weight (BW) 6.51 ± 0.64 kg, 21 days old) were selected, blocked by BW, and assigned to five dietary treatments with 12 replicate cages per treatment and one pig per metabolic cage. “Pre-care period” and “late-care period” diets were formulated as starch-soybean meal-based diets that met the nutrient requirements established by the NRC (16) for pigs weighing 7–11 kg and 11–25 kg, respectively.

High-amylose maize starch (High-Maize 1043, National Starch Industry, Shanghai, China) and waxy maize starch (food market, Hengyang, China) were blended to create diets with differing dietary amylose/amylopectin ratios (DAR) of 0.00, 0.20, 0.40, 0.60, or 0.80, respectively (Supplementary Table 1). According to the manufacturer’s instructions, the amylose and amylopectin contents were determined using their commercial assay kits (l-AMYL, Megazyme International Ireland Ltd., Wicklow, Ireland).

Pigs were fed and provided food *ad libitum*. Starting on day 15, the feed was gradually transitioned from the “pre-care period” diet to the “late-care period” diet over seven consecutive days, with ratios adjusted as follows: 80:20, 60:40, 50:50, 40:60, 30:70, 20:80, and 0:100. The experiment lasted 28 days.

On day 28, 12 h before slaughter, six pigs from each treatment group received intraperitoneal injections of 100 μg/kg BW lipopolysaccharides (LPS, from *Escherichia coli* O55:B5, Sigma Chemical Inc., St Louis, MO, USA, L2880), whereas another six pigs were administered an equivalent amount of sterile saline.

### 2.2 Sample collection

On day 15 before feed transition and on day 29, after LPS stress, blood samples were collected from the jugular vein and centrifuged at 3,000×*g* at 4°C for 10 min to separate serum. Piglets were euthanized via an intravenous injection of 40 mg/kg BW sodium pentobarbital solution into the jugular vein. The middle right lobe of the liver and chyme from the cecal and colon were collected using sterile scissors and tweezers and immediately stored at −80°C for further study.

### 2.3 Analysis of serum biochemical variables

Serum samples were thawed, and the concentration of total protein (TP), albumin (ALB), alanine transaminase (ALT), aspartate aminotransferase (AST), blood urea nitrogen (BUN), creatinine (CREA), glucose (GLU), triglyceride (TG), total cholesterol (CHOL), amylase (AMS), hepatic lipase (LIPC) was measured using commercial kits in accordance with the manufacturer’s instructions (Jiancheng Bioengineering Institute, Nanjing, China) at the TBA-120FR Automatic Biochemistry Radiometer (Hitachi Co., Tokyo, Japan). Serum immunoglobulin G and M (IgG, IgM) concentrations were determined using a commercial ELISA kit (Jiancheng Bioengineering Institute, Nanjing, China) according to the manufacturer’s instructions.

### 2.4 Analysis of AA contents in serum and liver

Samples were prepared for amino acid analysis as follows:

For serum, 600 μL of serum was mixed with an equivalent volume of 8% sulfosalicylic acid and incubated for 1 h at 4°C. Then, the mixture was then centrifuged at 10,000 rpm for 10 min at 4°C to collect supernatants.

For liver samples, approximately 0.1 g of ground, freeze-dried liver tissue was hydrolyzed in 10 mL of 6 mol/L hydrochloric acid solution at 110°C for 24 h. The hydrolyzed solution was diluted with distilled water to a final volume of 100 mL, and 1 mL of the supernatant was used for further analysis.

Amino acid concentrations were measured using an amino acid analyzer (L-8900, Hitachi, Japan) after both the serum supernatant and hepatic diluent solution were filtered through a 0.45-μm membrane.

### 2.5 Assay of hepatic antioxidant enzyme activities

Hepatic tissue samples were ground in liquid nitrogen, homogenized in saline, and centrifuged at 3,000×*g* 4°C for 10 min. The concentrations of malonaldehyde (MDA) and the activities of superoxide dismutase (SOD), including coper-zinc SOD (CuZn-SOD) and manganese SOD (Mn-SOD), glutathione peroxidase (GSH-PX/GPx), and total antioxidant capacity (T-AOC), were analyzed using commercial kits (Jiancheng Bioengineering Institute, Nanjing, China) according to the manufacturer’s instructions.

### 2.6 RNA extraction and real-time quantitative PCR

Total RNA was isolated from the liver using RNAiso Plus (TaKaRa), and then reverse transcription reactions were performed using an RT reagent kit (TaKaRa). All the procedures were carried out as described by the manufacturer’s protocol. The quantity and quality of RNA were determined using the NanoDrop ND-2000 spectrophotometer system (ThermoFisher Scientific). Primers were designed to assay genes related to antioxidant function (Supplementary Table 2). The β-actin was used as a reference gene. Real-time RT-PCR for target genes was performed on the MyIQ instrument (Bio-Rad, Hercules, California) using *SYBR* Green quantitative PCR mix (TaKaRa).

### 2.7 Analysis of mineral element contents in the liver

Macro and microelements (Ca, S, Mg, Cu, Fe, Mn, Zn) were analyzed using an analytical method described earlier (17). Briefly, liver samples (5.00 ± 0.20 g) were weighed in triplicate and digested using a mixture of acid (25 mL of HNO_3_: HClO_4_ in a volume ratio of 4:1) following heating (80°C for 60 min; 120°C for 30 min; and 180°C for 30 min). Samples were dried at 260°C and redissolved in 5 mL of 1% HNO_3_. The solution was then transferred to a 25-mL volumetric flask and filled with 1% HNO_3_. Finally, samples’ target elements (Cu, Fe, Mn, and Zn) were determined on ICP-OES (Varian 720ES Agilent, Santa Clara, CA, USA) for confirmation with standard references.

### 2.8 Analysis of indole and skatole contents in chyme

Indole and skatole (3-methylindole) of luminal contents in the cecal and colon were analyzed as described previously on high-performance liquid chromatography (HPLC, Agilent 1260, Agilent Technologies Inc., USA) (18). Briefly, luminal contents from the cecum and colon were weighed (1.000 g) and extracted overnight using 8 mL of 0.4 mol/L perchloric acid. Then, the suspension was obtained by centrifugation at 8,000 r/min for 10 min at 4°C. Subsequently, 1 mL of the supernatant was mixed with 1 mL of sodium hydroxide (2 mol/L, pH 10.6) saturated sodium bicarbonate buffer solution. Then, 1 mL of dansulfonyl chloride (10 mg/mL acetone solution) was added to the mixture. The solution was incubated in a water bath at 40°C in a dark environment for 30 min, followed by the addition of 1 mL of 5% ammonia water. Subsequently, 3 mL of anhydrous ether was used to extract the solution. The anhydrous ether layer was dried, dissolved in 1 mL of methanol, filtered through a 0.22-μm syringe filter membrane, and analyzed using the appropriate analytical instrument.

### 2.9 Statistics analysis

Gene expression data from replicate measurements within the same RNA extraction were averaged and analyzed using the Livak (19) method. One-way ANOVA followed by Duncan’s multiple range test was conducted using SAS 8.0 to evaluate significant differences in serum indices based on DAR on Day 15. Data from D29 were analyzed using two-way ANOVA, with “stress” (saline or LPS), dietary DAR, and their interaction as factors.

Duncan’s multiple range test was used to compare differences among the various DAR groups. SAS’s PROC REG procedure (with stepwise selection at a *p*-value of <0.15) was used to analyze potential linear or quadratic regressions between dietary DAR and serum or hepatic indices in non-stressed piglets. Data were expressed as least squares mean (Lsmeans) ± SEM. A *p-*value of <0.05 was considered statistically significant, while 0.05 < *p* < 0.10 indicated a statistically significant trend.

## 3 Results

### 3.1 Serum biochemical variables at D15

DAR did not affect serum ALT, AST, LIPC, TP, ALB, UREA, CREA, or TC on D15 (Table 1). However, piglets in the DAR 0.00 and 0.60 groups exhibited higher serum AMS activity than those in the DAR 0.40 group (*p* < 0.05). Serum GLU levels were significantly higher in the DAR 0.80 group than in the other groups (*p* < 0.05). Serum TG levels were higher in the DAR 0.20 group compared to DAR 0.00 and 0.40 (*p* < 0.05). Furthermore, serum GLU levels increased quadratically with increasing DAR (*p* < 0.05).

**Table 1:** Effects of dietary amylose/amylopectin ratio on serum biochemical index on day 15 in weaned piglets.

Items^1^	0.00	0.20	0.40	0.60	0.80	SEM	*p* value	*P_L_*	*P_Q_*
ALT, U/L	34.48	36.59	42.79	44.38	39.13	1.58	0.264	0.142	NS
AST, U/L	55.73	62.25	55.67	53.67	51.00	2.50	0.693	NS	NS
LIPC, U/L	4.22	4.56	3.93	4.55	4.13	0.12	0.421	NS	NS
AMS, U/L	2,642^A^	2,230^AB^	2,022^B^	2,493^A^	2,226^AB^	63.79	0.031	NS	NS
TP, g/L	47.22	49.51	45.60	45.97	45.12	0.68	0.260	−0.108	−0.102
ALB, g/L	25.59	26.73	24.53	26.29	25.70	0.52	0.725	NS	NS
UREA, mmol/L	4.54	5.13	4.83	4.48	4.73	0.22	0.895	NS	NS
CREA, umol/L	60.00	63.92	66.92	63.75	63.00	1.60	0.762	NS	NS
GLU, mmol/L	4.68^B^	4.73^B^	4.53^B^	4.58^B^	5.32^A^	0.08	0.027	0.070	0.017
TG, mmol/L	0.45^B^	0.60^A^	0.40^B^	0.47^AB^	0.53^AB^	0.02	0.023	NS	NS
TC, mmol/L	1.87	2.07	1.87	2.02	2.05	0.05	0.405	NS	NS

1 ALT, alanine transaminase; AST, aspartate aminotransferase, LIPC, hepatic lipase; AMS, amylase; TP, total protein; ALB, albumin; UREA, urea nitrogen; CREA, creatinine; GLU, glucose; TG, triglyceride; TC, total cholesterol; SEM, standard error of the mean; L, Q represent linear, quadratic response to increasing dietary amylose/amylopectin ratio. ^A-B^Values within a row with different superscripts differ significantly at *p* < 0.05. NS means a *p*-value of > 0.15.

### 3.2 Serum-free amino acids at D15

Serum essential amino acids on D15 were not significantly affected by DAR (*p* > 0.05), except for serum Arg concentrations, which were higher in the DAR 0.40 and 0.60 groups compared to DAR 0.00 (*p* < 0.05) (Table 2). Serum non-essential amino acids, such as tyrosine (Tyr), glutamate (Glu), glycine (Gly), serine (Ser), cysteine (Cys), taurine (Tau), urea, hydroxylysine (Hylys), ornithine (Orn), β-alanine (β-Ala), β-aminoisobutyric acid (β-AiBA), 1-methylhistidine (1Mehis), 3-methylhistidine (3Mehis), and hydroxy-L-proline (Hypro), were not significantly affected by DAR (*p* > 0.05).

**Table 2:** Effects of dietary amylose/amylopectin ratio on serum free amino acid concentration on day 15 in weaned piglets, ng/20 uL.

Items, full name	Items, abbreviation	0.00	0.20	0.40	0.60	0.80	SEM	*p* value	*P_L_*	*P_Q_*
Essential amino acid	–									
Lysine	Lys	418.65	488.86	441.79	413.45	432.75	19.48	0.752	NS	NS
Methionine	Met	118.90	131.43	122.62	134.92	147.40	4.92	0.403	NS	NS
Histidine	His	137.54	154.34	126.72	140.73	144.51	3.44	0.154	NS	NS
Isoleucine	Ile	200.15	227.27	203.17	206.78	212.50	5.90	0.623	NS	NS
Leucine	Leu	272.47	305.75	255.92	261.08	252.83	7.08	0.126	NS	NS
Phenylalanine	Phe	206.62	219.99	197.31	202.84	203.49	4.55	0.590	−0.097	−0.087
Valine	Val	368.39	402.46	347.66	370.04	365.57	8.80	0.400	NS	NS
Threonine	Thr	131.98	174.13	138.68	140.79	160.39	8.79	0.537	NS	NS
Arginine	Arg	407.26^B^	471.80^AB^	540.05^A^	528.63^A^	434.36^AB^	15.44	0.037	NS	NS
Non-essential amino acid	–									
Tyrosine	Tyr	156.28	177.26	152.29	166.32	187.46	5.92	0.314	0.085	0.072
Alanine	Ala	648.46^B^	781.34^AB^	710.91^B^	710.52^B^	860.09^A^	21.02	0.029	0.028	0.023
Aspartate	Asp	100.80^A^	102.90^A^	91.02^AB^	80.77^B^	82.94^B^	2.51	0.019	−0.002	−0.003
Glutamate	Glu	734.33	747.36	586.50	639.88	820.27	29.13	0.101	NS	NS
Glycine	Gly	810.43	773.85	811.29	758.24	875.34	30.42	0.773	NS	NS
Serine	Ser	173.97	188.12	183.17	158.89	195.41	6.19	0.390	NS	NS
Cysteine	Cys	93.28	119.84	101.66	105.77	109.84	2.94	0.082	NS	NS
Proline	Pro	290.28^B^	370.19^A^	412.60^A^	400.86^A^	453.46^A^	12.43	0.003	0.000	0.001
Taurine	Tau	237.35	242.97	214.22	191.53	216.51	5.98	0.065	−0.035	−0.078
Urea	Urea	1,971	2,219	1,948	1,891	1,967	82.46	0.750	NS	NS
Sarcosine	Sar	5.21^B^	9.82^B^	31.15^A^	14.98^B^	10.27^B^	2.30	0.008	NS	NS
Citrulline	Cit	132.51^C^	161.98^AB^	132.65^C^	141.87^BC^	170.41^A^	4.01	0.009	0.078	0.046
Anserine	Ans	20.60^A^	1.61^B^	2.51^B^	3.19^B^	1.80^B^	1.83	0.008	−0.014	−0.079
Carnosine	Car	40.20^B^	48.23^B^	94.83^A^	110.27^A^	52.58^B^	5.76	0.001	0.074	NS
Cystathionine	Cysthi	13.07^B^	16.78^AB^	15.94^AB^	15.59^AB^	18.45^A^	0.55	0.054	0.021	0.031
Hydroxylysine	Hylys	14.74	17.63	16.17	14.23	17.52	0.67	0.383	NS	NS
Ornithine	Orn	118.98	136.44	118.74	117.85	140.36	5.55	0.537	NS	NS
a-Aminoadipic acid	a-AAA	118.00^AB^	91.46^B^	33.50^C^	49.56^C^	133.74^A^	5.34	<0.0001	NS	NS
a-Amino-n-butyric acid	a-ABA	12.61^C^	20.14^AB^	16.63^BC^	17.72^ABC^	23.40^A^	0.94	0.012	0.009	0.010
β-Alanine	β-Ala	37.47	33.59	36.23	36.23	35.88	1.82	0.975	NS	NS
β-Aminoisobutyric acid	β-AiBA	3.49	6.82	6.70	8.78	7.22	0.57	0.085	0.029	0.100
γ-Aminobutyric acid	γ-ABA	0.97^B^	1.133^B^	1.11^B^	1.88^A^	0.96^B^	0.11	0.043	NS	NS
Ethanolamine	EOHNH2	10.25^B^	12.49^A^	9.16^B^	9.32^B^	9.18^B^	0.23	<0.0001	−0.005	−0.004
1-Methylhistidine	1Mehis	61.51	57.55	55.54	49.90	56.91	2.41	0.666	NS	NS
3-Methylhistidine	3Mehis	33.20	32.72	30.37	33.34	34.29	1.71	0.963	NS	NS
Hydroxy-L-proline	Hypro	181.21	156.19	137.98	132.13	142.29	6.13	0.112	−0.025	−0.092
Sum	Sum	167.40	181.49	161.25	157.77	168.82	4.86	0.586	NS	NS

^A-C^Values within a row with different superscripts differ significantly at *p* > 0.05. NS means a *p* < 0.15. L, Q represents a linear, quadratic response to increasing dietary amylose/amylopectin ratio (DAR).

Piglets in the DAR 0.80 group exhibited significantly higher serum alanine (Ala) and citrulline (Cit) concentrations compared to those in the DAR 0.00, 0.40, and 0.60 groups (*p* < 0.05), but these levels were not significantly different from those in the DAR 0.20 group (*p* > 0.05). Serum aspartate (Asp) concentrations were significantly lower in the DAR 0.80 and 0.60 groups compared to DAR 0.00 and 0.20 (*p* < 0.05). Piglets in the DAR 0.40 and 0.60 groups exhibited significantly higher serum carnosine (Car) concentrations than those in other groups (*p* < 0.05).

Serum *α*-amino-butyric acid (α-ABA) concentrations were significantly higher in the DAR 0.80 group compared to DAR 0.00 and 0.40 (*p* < 0.05). Serum proline (Pro) levels were significantly lower in the DAR 0.00 group than in all other groups (*p* < 0.05). Serum ethanolamine (EOHNH2) levels were significantly higher in the DAR 0.20 group compared to all other groups (*p* < 0.05). Piglets in the DAR 0.40 group had the highest serum sarcosine (Sar) concentrations among all groups (*p* < 0.05), while those in the DAR 0.60 group had the highest serum *γ*-aminobutyric acid (γ-ABA) concentrations (*p* < 0.05). Serum *α*-aminoadipic acid (a-AAA) concentrations in the DAR 0.80 group were significantly higher than in DAR 0.20, 0.40, and 0.60 (*p* < 0.05).

REG analysis showed that serum Phe tended to decrease linearly with increasing DAR (*p* = 0.097), while serum Tyr (*p* = 0.085) and Car (*p* = 0.074) tended to increase linearly with increasing DAR. Serum Ala, Pro, Cysthi, α-ABA, β-AiBA increased linearly with increasing DAR (*p* < 0.05), whereas serum Asp, Tau, Ans, EOHNH2, and Hypro decreased linearly with increasing DAR (*p* < 0.05).

### 3.3 Serum biochemical variables at D29

Different DAR diets showed no significant differences in serum ALT, AST, AMS, TP, ALB, UREA, CREA, GLU, TG, and IgM levels at D29 (Table 3, *p* > 0.05). Serum LIPC was significantly higher in the DAR 0.60 group compared to the DAR 0.00 and 0.20 groups (*p* < 0.05). Piglets in the DAR 0.60 and 0.80 groups exhibited higher serum TC levels than those in the DAR 0.40 group (*p* < 0.05). Serum IgG concentration was significantly higher in the DAR 0.80 group than in the other groups (*p* < 0.05). Serum AST decreased progressively with increasing DAR (*p* < 0.05). Serum TP decreased, whereas IgG increased linearly with increasing DAR (*p* < 0.05). Serum TC tended to increase linearly with increasing DAR (*p* = 0.066).

**Table 3:** Effects of dietary amylose/amylopectin ratio on serum biochemical index on day 29 in weaned piglets under LPS stress.

Items^1^	0.00		0.20		0.40		0.60		0.80		SEM	*p* value			*P_L_*	*P_Q_*
	LPS^2^	SAL	LPS	SAL	LPS	SAL	LPS	SAL	LPS	SAL		*P*_DAR_	*P*_stress_	*P*_D*S_		
ALT, U/L	33.98	41.86	38.83	39.78	42.48	44.20	39.88	46.48	31.42	41.98	1.28	0.324	0.031	0.701	NS	NS
AST, U/L	67.83	72.80	89.50	69.00	80.17	77.17	110.67	56.33	99.67	54.50	3.72	0.853	0.002	0.055	−0.065	−0.045
LIPC, U/L	3.55^C^	3.80	3.75^BC^	4.63	4.30^AB^	5.22	4.58^A^	6.02	4.15^ABC^	4.65	0.16	0.027	0.014	0.798	0.137	NS
AMS, U/L	2,487	2,248	2,153	2,454	1,950	2,429	2,585	2,370	2,406	2,593	81.08	0.712	0.519	0.528	NS	NS
TP, g/L	43.63	55.16	44.58	52.50	43.13	43.27	44.82	48.22	45.82	43.40	0.94	0.192	0.030	0.131	−0.021	−0.046
ALB, g/L	23.72	21.46	21.92	25.22	21.68	21.35	25.65	23.98	23.33	22.60	0.60	0.483	0.774	0.605	NS	NS
UREA, mmol/L	6.17	5.38	6.57	5.58	9.03	5.65	5.77	5.02	6.63	5.18	0.36	0.460	0.039	0.728	NS	NS
CREA, umol/L	91.33	57.20	96.33	63.83	87.50	64.83	107.00	60.33	100.67	61.83	2.26	0.670	0.000	0.521	NS	NS
GLU, mmol/L	4.43	4.90	4.17	5.23	3.83	5.22	3.88	4.97	3.37	5.33	0.12	0.869	0.000	0.419	NS	NS
TG, mmol/L	0.73	0.46	0.66	0.43	0.56	0.48	0.71	0.46	0.71	0.50	0.02	0.664	0.000	0.638	NS	NS
TC, mmol/L	2.14^AB^	2.01	2.12^AB^	2.21	1.78^B^	2.03	2.11^A^	2.30	2.28^A^	2.37	0.04	0.014	0.238	0.675	0.066	0.061
IgG, g/L	0.37^E^	0.87	1.09^D^	6.99	5.57^C^	7.52	6.74^B^	17.19	16.41^A^	12.85	0.26	<0.0001	<0.0001	<0.0001	<0.0001	<0.0001
IgM, g/L	0.66	0.85	0.51	0.51	0.54	0.55	0.47	0.77	0.68	0.54	0.03	0.146	0.259	0.171	NS	NS

1 ALT, alanine transaminase; AST, aspartate aminotransferase, LIPC, hepatic lipase; AMS, amylase; TP, total protein; ALB, albumin; UREA, urea nitrogen; CREA, creatinine; GLU, glucose; TG, triglyceride; TC, total cholesterol; IgG, immunoglobulin G; IgM, immunoglobulin M.

2 LPS, lipopolysaccharide, SAL, saline, SEM, standard error of the mean, DAR, dietary amylose/amylopectin ratio. L, Q represents a linear, quadratic response to increasing dietary amylose/amylopectin ratio. Only data from non-stress piglets were analyzed in this REG procedure.

^A-EValues within a row with different superscripts differ significantly at p < 0.05. NS means a p-value of > 0.15.^

As shown in Table 3, LPS stimulation significantly increased serum AST, UREA, CREA, and TG levels but decreased serum ALT, LIPC, TP, and GLU concentrations compared to controls (*p* < 0.05). Serum AMS, ALB, TC, and IgM concentrations were not significantly affected by LPS administration (*p* > 0.05).

A significant interaction between LPS and DAR was found for serum IgG concentrations (*p* < 0.01). In LPS-exposed animals, serum IgG concentration significantly increased in the DAR 0.80 group, whereas it decreased in the other groups after LPS administration. Additionally, a two-way interaction between LPS stress and DAR was observed for serum AST concentrations (*p* = 0.055). Serum AST levels tended to decrease in the DAR 0.00 group after the LPS challenge compared to the control group. In contrast, serum AST levels increased following the LPS challenge in the other DAR groups after the LPS challenge in other groups.

### 3.4 Serum-free amino acids at D29

Serum essential amino acid concentrations were not significantly affected by DAR (*p* > 0.05), except for serum Arg, which was higher in the DAR 0.80 group compared to the DAR 0.00, 0.20, and 0.40 groups (Table 4, *p* < 0.05). Similarly, serum non-essential amino acids were not affected by DAR (*p* > 0.05), except for Pro, Sar, Cit, Ans, Hylys, β-AiBA, and Hypro (*p* < 0.05). Serum Pro was significantly higher in the DAR 0.60 group than in the DAR 0.80, 0.40, and 0.00 groups (*p* < 0.05). Serum concentrations of Sar, Cit, Ans, and Hylys in the DAR 0.80 group were significantly higher than in the DAR 0.00 group (*p* < 0.05).

**Table 4:** Effects of dietary amylose/amylopectin ratio on serum-free AA concentration on day 29 in weaned piglets under LPS stress, ng/20 uL.

Items^1^	0.00		0.20		0.40		0.60		0.80		SEM	*p* value			*P_L_*	*P_Q_*
	LPS^2^	SAL	LPS	SAL	LPS	SAL	LPS	SAL	LPS	SAL		*P*_DAR_	*P*_stress_	*P*_D*S_		
Lys	290.83	461.73	299.34	439.69	344.25	383.30	289.37	517.96	362.64	529.83	14.10	0.332	<0.0001	0.310	NS	0.139
Met	88.53	109.98	71.44	145.46	80.73	101.33	67.04	168.53	63.65	148.75	3.99	0.290	<0.0001	0.004	0.092	0.104
His	111.83	114.56	111.21	124.90	129.58	116.57	97.37	126.11	118.01	128.32	3.08	0.662	0.175	0.301	NS	NS
Ile	189.06	241.42	148.19	298.79	172.41	234.18	148.91	281.43	130.05	280.38	6.91	0.883	<0.0001	0.060	NS	NS
Leu	209.84	248.70	186.51	270.96	222.90	213.75	180.88	240.34	179.25	247.79	5.85	0.789	0.000	0.119	NS	NS
Phe	190.14	191.68	158.86	228.87	205.48	185.08	159.12	232.94	176.52	242.75	5.15	0.811	0.001	0.011	0.085	0.069
Val	321.02	375.51	292.89	450.46	350.52	357.90	279.32	407.13	273.61	427.02	9.16	0.886	<0.0001	0.045	NS	NS
Thr	88.26	93.72	85.80	126.89	96.80	96.43	86.83	121.27	83.86	122.10	3.80	0.715	0.003	0.272	NS	NS
Arg	328.56^B^	365.26	300.14^B^	430.07	310.30^B^	417.24	275.51^AB^	537.74	347.81^A^	606.33	12.69	0.015	<0.0001	0.028	0.001	0.001
NEAA																
Tyr	175.17	181.98	149.26	224.12	157.65	170.40	131.81	229.11	133.36	256.84	5.27	0.437	<0.0001	0.003	0.022	0.011
Ala	817.28	732.17	881.35	834.48	961.51	669.89	1,219.58	817.30	903.89	703.50	27.71	0.060	0.001	0.248	NS	NS
Asp	64.50	78.11	59.85	92.65	73.18	78.19	54.64	83.10	60.92	80.89	2.61	0.872	0.000	0.456	NS	NS
Glu	616.87	639.59	526.48	793.71	584.36	642.12	495.57	735.61	553.77	661.64	23.35	0.955	0.005	0.375	NS	NS
Gly	785.89	620.18	722.03	948.18	874.09	789.05	1,016	795.10	668.99	775.45	27.50	0.128	0.614	0.066	NS	NS
Ser	169.54	137.75	150.71	207.84	179.11	156.57	206.91	180.88	150.52	165.47	4.97	0.089	0.869	0.033	NS	NS
Cys	104.91	81.56	99.86	76.33	113.40	94.96	119.98	93.51	131.90	94.06	3.12	0.092	0.000	0.894	0.141	NS
Pro	287.69^C^	325.81	350.11^AB^	477.26	350.40^BC^	355.05	411.45^A^	503.95	333.15^BC^	405.19	10.57	0.001	0.003	0.408	NS	NS
Other AA																
Tau	170.19	207.61	169.97	192.20	181.97	162.60	156.89	163.98	172.31	186.49	4.70	0.405	0.197	0.427	NS	NS
Urea	2,985	2,251	2,747	2,300	2,445	2,480	2,459	2,121	2,912	2,307	65.11	0.502	0.002	0.410	NS	NS
Sar	5.03^C^	6.57	23.50^A^	27.01	14.50^B^	14.04	31.95^A^	34.50	29.80^A^	30.71	1.06	<0.0001	0.531	0.992	0.003	0.010
Cit	166.38^B^	136.89	186.49^AB^	164.00	190.54^AB^	154.22	182.22^AB^	174.65	221.01^A^	190.20	4.85	0.024	0.012	0.900	0.052	0.037
Ans	2.94^BC^	2.37	0.52^C^	0.00	4.11^ABC^	5.22	5.44^AB^	7.60	11.54^A^	7.16	0.73	0.003	0.766	0.677	0.033	0.054
Car	47.58	30.79	37.03	38.89	44.61	39.59	53.22	26.42	43.69	32.99	1.57	0.926	0.001	0.057	NS	NS
Cysthi	20.40	14.14	17.15	20.00	17.05	15.95	18.66	17.41	17.04	14.88	0.74	0.787	0.289	0.450	NS	NS
Hylys	12.04^B^	13.48	9.06^B^	11.64	11.63^B^	13.00	11.80^B^	13.03	18.29^A^	18.69	0.67	0.005	0.302	0.991	NS	0.079
Orn	155.05	232.42	173.61	243.92	171.88	211.13	165.96	256.64	197.85	276.09	5.67	0.100	<0.0001	0.674	0.031	0.028
a-AAA	149.35	109.72	131.54	164.00	151.77	137.55	164.84	157.76	170.94	158.45	5.77	0.357	0.490	0.420	NS	NS
a-ABA	16.66	21.25	17.80	27.98	17.62	17.92	12.13	24.97	14.23	26.31	1.06	0.582	0.001	0.288	NS	NS
β-Ala	57.39	31.15	34.28	30.19	41.16	26.63	45.87	39.06	53.16	35.74	1.89	0.115	0.001	0.389	NS	NS
β-AiBA	5.80^A^	7.28	0.92^B^	1.72	3.10^B^	1.59	4.18^A^	8.38	8.11^A^	4.98	0.42	0.000	0.676	0.117	NS	NS
γ-ABA	2.09	1.69	1.50	1.66	2.78	1.25	1.45	3.92	3.11	3.25	0.26	0.304	0.748	0.193	0.137	0.149
EOHNH2	9.86	10.81	10.18	9.17	10.02	7.62	10.76	9.66	13.45	9.24	0.40	0.368	0.057	0.361	NS	NS
1Mehis	71.09	42.86	47.79	62.23	65.33	55.25	59.19	64.74	56.45	58.50	2.37	0.891	0.495	0.068	NS	NS
3Mehis	13.65	33.01	12.85	17.08	24.80	19.76	13.94	16.75	20.60	18.02	1.32	0.167	0.160	0.055	NS	NS
Hypro	161.69^B^	85.02	155.94^A^	161.40	153.41^AB^	155.79	199.73^A^	138.12	142.28^AB^	146.45	4.20	0.019	0.004	0.003	NS	NS

1 EAA, Essential amino acid; NEAA, Non-essential amino acid; Lys, Lysine; Met, Methionine; His, Histidine; Ile, Isoleucine; Leu, Leucine; Phe, Phenylalanine; Val, Valine; Thr, Threonine; Arg, Arginine; Tyr, Tyrosine; Ala, Alanine; Asp, Aspartate; Glu, Glutamate; Gly, Glycine; Ser, Serine; Cys, Cysteine; Pro, Proline; Tau, Taurine; Sar, Sarcosine; Cit, Citrulline; Ans, Anserine; Car, Carnosine; Cysthi, Cystathionine; Hylys, Hydroxylysine; Orn, Ornithine; a-AAA, a-Aminoadipic acid; a-ABA, a-Amino-butyric acid; b-Ala, β-Alanine; b-AiBa, β-Aminoisobutyric acid; γ-ABA, γ-Aminobutyric acid; EOHNH2, Ethanolamine; 1Mehis, 1-Methylhistidine; 3-Mehis, 3-Methylhistidine; Hypro, Hydroxy-L-proline.

^2LPS, lipopolysaccharide, SAL, saline, SEM, standard error of the mean, L, Q represents a linear, quadratic response to increasing dietary amylose/amylopectin ratio. Only data from non-stress piglets were analyzed in this REG procedure.^

^A-CValues within a row with different superscripts differ significantly at p < 0.05. NS means a p-value of > 0.15.^

Pigs from the DAR 0.00, 0.60, and 0.80 groups exhibited significantly higher serum β-AiBA levels than those from the DAR 0.20 and 0.40 groups (*p* < 0.05). Serum Hypro concentrations were significantly higher in the DAR 0.20 and 0.60 groups than in the DAR 0.00 group (*p* < 0.05). Serum essential amino acid concentrations decreased after LPS stress (*p* < 0.05), except for His, which was not affected by LPS stress (*p* > 0.05).

Serum concentrations of Tyr, Ala, Asp, Glu, Cys, Pro, urea, Cit, Car, Orn, α-ABA, β-Ala, and Hypro were significantly affected by LPS stress (*p* < 0.05).

A significant interaction effect between DAR and LPS was observed for serum Met, Phe, Val, Arg, Tyr, Ser, and Hypro (*p* < 0.05). Serum Phe levels increased after LPS stress in the DAR 0.40 group but decreased in the other groups (*p* < 0.05). Serum Met and Val concentrations remained stable after LPS stress in the DAR 0.00 and 0.40 groups but decreased in the other groups (*p* < 0.05). Serum Arg and Tyr levels were stable after LPS stress in the DAR 0.00 group but decreased in the other groups (*p* < 0.05). Serum Ser concentrations decreased after LPS stress in the DAR 0.20 and 0.80 groups but increased in the other groups (*p* < 0.05). Serum Hypro levels increased after LPS stress in the DAR 0.00 and 0.60 groups but remained stable in the other groups (*p* < 0.05).

Serum Arg (*p* < 0.01), Tyr (*p* < 0.05), Sar (*p* < 0.01), Ans (*p* < 0.05), and Orn (*p* < 0.05) increased linearly with increasing DAR, while serum Cit exhibited a progressive increase with increasing DAR (*p* < 0.05).

### 3.5 Amino acid contents in liver

There was no significant effect of DAR on all tested hepatic amino acid concentrations (*p* > 0.05), except for hepatic Cys, which was significantly lower in the DAR 0.80 group compared to the DAR 0.00 and 0.20 groups (*p* < 0.05) (Table 5). The LPS challenge resulted in increased concentrations of all tested amino acid concentrations in the liver compared to the control group (*p* < 0.05).

**Table 5:** Effects of dietary amylose/amylopectin ratio on amino acid composition in the liver of weaned piglets under LPS stress, μg/100 mg.

Items^1^	0.00		0.20		0.40		0.60		0.80		SEM	*p* value			*P_L_*	*P_Q_*
	LPS^2^	SAL	LPS	SAL	LPS	SAL	LPS	SAL	LPS	SAL		*P*_DAR_	*P*_stress_	*P*_D*S_		
Lys	3.20	2.60	2.79	2.97	3.11	2.52	3.18	2.32	2.87	2.42	0.05	0.526	<0.0001	0.057	−0.025	−0.023
Met	0.91	0.70	0.83	0.89	0.90	0.73	0.90	0.67	0.82	0.65	0.01	0.181	<0.0001	0.083	−0.045	−0.019
His	1.07	0.90	0.97	1.03	1.04	0.87	1.03	0.82	0.95	0.83	0.01	0.133	<0.0001	0.120	−0.024	−0.019
Ile	1.84	1.54	1.72	1.80	1.82	1.51	1.86	1.38	1.70	1.48	0.02	0.242	<0.0001	0.034	−0.036	−0.034
Leu	3.86	3.23	3.57	3.79	3.87	3.22	3.91	3.00	3.64	3.17	0.05	0.562	<0.0001	0.049	−0.078	−0.065
Phe	2.04	1.76	1.90	2.03	2.06	1.71	2.04	1.57	1.90	1.61	0.03	0.148	<0.0001	0.042	−0.012	−0.009
Val	2.36	2.01	2.21	2.31	2.32	1.91	2.36	1.78	2.15	1.87	0.03	0.129	<0.0001	0.062	−0.015	−0.017
Thr	1.98	1.66	1.81	1.93	1.99	1.61	1.99	1.51	1.85	1.55	0.03	0.317	<0.0001	0.031	−0.019	−0.015
Arg	2.77	2.29	2.57	2.67	2.78	2.22	2.80	2.05	2.59	2.14	0.04	0.320	<0.0001	0.059	−0.023	−0.021
Asp	4.00	3.36	3.69	3.88	4.03	3.29	4.04	3.08	3.76	3.18	0.05	0.414	<0.0001	0.046	−0.028	−0.023
Ser	2.11	1.79	1.92	2.05	2.10	1.72	2.12	1.61	1.96	1.66	0.03	0.311	<0.0001	0.037	−0.014	−0.012
Glu	6.11	5.14	5.65	5.87	6.21	5.06	6.19	4.75	5.79	4.88	0.07	0.419	<0.0001	0.041	−0.020	−0.015
Gly	2.48	2.14	2.28	2.42	2.47	2.08	2.52	1.891	2.29	2.02	0.03	0.378	<0.0001	0.047	−0.027	−0.030
Ala	2.76	2.48	2.68	2.82	2.86	2.40	2.94	2.23	2.71	2.33	0.04	0.464	<0.0001	0.056	−0.020	−0.020
Cys	0.32^A^	0.28	0.33^A^	0.32	0.33^AB^	0.22	0.36^AB^	0.19	0.29^B^	0.20	0.01	0.034	<0.0001	0.126	−0.002	−0.005
Tyr	1.39	1.10	1.34	1.46	1.52	1.25	1.51	1.15	1.451	1.17	0.02	0.265	<0.0001	0.051	NS	NS
NH3	0.68	0.57	0.64	0.62	0.67	0.53	0.67	0.51	0.60	0.50	0.01	0.065	<0.0001	0.406	−0.005	−0.005
Pro	2.01	1.71	1.85	1.99	2.03	1.68	2.08	1.58	1.93	1.64	0.03	0.675	<0.0001	0.052	−0.052	−0.044

1 Lys, Lysine; Met, Methionine; His, Histidine; Ile, Isoleucine; Leu, Leucine; Phe, Phenylalanine; Val, Valine; Thr, Threonine; Arg, Arginine; Asp, Aspartate; Ser, Serine; Glu, Glutamate; Gly, Glycine; Ala, Alanine; Cys, Cysteine; Tyr, Tyrosine; Pro, Proline.

2 LPS, lipopolysaccharide, SAL, saline, SEM, standard error of the mean, DAR, dietary amylose/amylopectin ratio. L, Q represents a linear, quadratic response to increasing dietary amylose/amylopectin ratio. Only data from non-stress piglets were analyzed in this REG procedure.

^A-BValues within a row with different superscripts differ significantly at p < 0.05. NS means a p-value of > 0.15.^

A significant interaction between LPS and DAR was observed for hepatic Ile, Leu, Phe, Thr, Asp, Ser, Glu, and Gly concentrations (*p* < 0.05). In animals fed the DAP 0.20, these amino acid concentrations decreased 12 h after LPS exposure. However, their concentrations increased in animals from other dietary groups following LPS stress.

### 3.6 Antioxidant function of liver

The concentration of hepatic MDA was significantly higher in the DAR 0.20 group compared to the DAR 0.00, 0.40, and 0.80 groups (*p* < 0.05) (Table 6). The activity of T-AOC was not affected by DAR or LPS stress (*p* > 0.05). The activity of GSH-PX in the DAR 0.20 group was higher than in the DAR 0.40, 0.60, and 0.80 groups (*p* < 0.05). It increased after LPS stress (*p* < 0.05). T-SOD activity in DAR 0.00 was higher than that in DAR 0.20, 0.40, and 0.80 (*p* < 0.05). After LPS stress, hepatic T-SOD activity decreased in the DAR 0.40 group but increased in the DAR 0.00 and 0.80 groups (*p* < 0.05). The activity of Cu-SOD was lower in the DAR 0.20 group compared to the other groups (*p* < 0.05) and increased linearly with rising DAR (*p* < 0.05).

**Table 6:** Effects of dietary amylose/amylopectin ratio on antioxidant function in the liver of weaned piglets under LPS stress.^1^

Items^1^	0.00		0.20		0.40		0.60		0.80		SEM	*p* value			*P_L_*	*P_Q_*
	LPS^2^	SAL	LPS	SAL	LPS	SAL	LPS	SAL	LPS	SAL		*P*_DAR_	*P*_stress_	*P*_D*S_		
Activities/concentration																
MDA, nmol/mg pro	0.83^C^	1.00	4.57^A^	3.60	2.03^BC^	2.01	2.91^AB^	3.39	1.69^BC^	2.15	0.20	0.000	0.958	0.797	NS	NS
T-AOC, mmol/mg pro	61.30	71.64	71.57	108.91	73.33	89.47	121.65	74.43	111.92	73.52	5.12	0.379	0.685	0.074	NS	NS
GSH-PX, U/mg pro	1,385^AB^	576.11	1,700^A^	805.25	585.06^C^	187.56	468.24^C^	172.90	748.88^BC^	422.90	73.75	0.001	0.001	0.557	−0.107	NS
T-SOD, U/mg pro	1,102^A^	358.90	54.75^C^	55.93	172.82^BC^	347.69	527.17^AB^	513.93	401.35^BC^	278.50	46.51	0.000	0.133	0.029	0.115	NS
Cu-SOD, U/mg pro	497.18^A^	212.97	39.01^B^	40.47	181.45^A^	365.42	468.21^A^	459.46	321.30^A^	225.84	37.94	0.001	0.500	0.184	0.010	0.063
mRNA expression																
SOD1	2.51^A^	1.06	1.05^B^	0.77	0.71^B^	0.89	1.08^B^	1.27	0.82^B^	0.69	0.07	0.000	0.044	0.005	–	–
Nrf2	2.40^A^	1.05	1.34^B^	1.01	0.94^B^	1.27	1.30^AB^	1.37	0.83^B^	1.04	0.07	0.016	0.148	0.004	–	–
GPX1	2.95^A^	1.10	1.73^B^	0.50	0.92^B^	0.64	1.27^B^	1.05	1.48^B^	0.67	0.10	0.006	0.000	0.083	–	–
GCLC	1.80	1.05	1.11	0.82	1.04	1.45	1.03	2.02	0.90	1.23	0.08	0.167	0.396	0.014	–	–

1 MDA, malonaldehyde; T-SOD, total superoxide dismutase; CuZnSOD, cooper-zinc; Cu-SOD, cooper-zinc superoxide dismutase; GSH-PX, glutathione peroxidase; T-AOC, total antioxidant capacity; *GCLC,* glutamate-cysteine ligase catalytic subunit; *Nrf2,* nuclear factor, erythroid 2 like 2; *SOD1,* superoxide dismutase; *GPX1,* and glutathione peroxidase 1.

2 LPS, lipopolysaccharide, SAL, saline, SEM, standard error of the mean, DAR, dietary amylose/amylopectin ratio. L, Q represents a linear, quadratic response to increasing dietary amylose/amylopectin ratio. Only data from non-stress piglets were analyzed in this REG procedure.

^A-CValues within a row with different superscripts differ significantly at p < 0.05. NS means a p-value of > 0.15. – means not applicable.^

Gene expression related to antioxidant function, such as *SOD1*, nuclear translocation factors (*Nrf2*), and *GPX1* mRNA expressions, was affected by DAR, which was higher in DAR 0.00 than in other groups (*p* < 0.05). LPS stress caused an increase in the abundance of gene *GPX1* (*p* < 0.05). mRNA expression of *SOD1* decreased in DAR 0.40 and 0.60 after LPS stress but increased in other groups (*p* < 0.05). *Nrf2* mRNA expression decreased in the DAR 0.40, 0.60, and 0.80 groups after LPS stress but increased in the DAR 0.00 and 0.20 groups (*p* < 0.05). Gene of glutamate-cysteine ligase catalytic subunit (*GCLC*) expression in the DAR 0.40, 0.60, and 0.80 groups decreased after LPS stress but increased in the DAR 0.00 and 0.20 groups (*p* < 0.05).

### 3.7 Mineral element contents in liver

Mineral element concentrations such as Ca, Mg, and S were not affected by DAR (*p* > 0.05) (Table 7). The DAR 0.80 had higher Cu, Fe, Mn, and Zn concentrations than DAR 0.00, 0.20, and 0.40 (*p* < 0.05). The LPS stress caused an increase in Mg and S concentrations (*p* < 0.05). Cu concentration (*p* < 0.01) increased after LPS stress in DAR 0.00 and 0.80 but decreased in other groups after LPS stress. Hepatic Cu, Fe, Mn, and Zn concentrations increased linearly with increasing DAR (*p* < 0.01).

**Table 7:** Effects of dietary amylose/amylopectin ratio on mineral element concentrations in the liver of weaned piglets under LPS stress.^1^

Items, mg/g	0.00		0.20		0.40		0.60		0.80		SEM	*p* value			*P_L_*	*P_Q_*
	LPS^1^	SAL	LPS	SAL	LPS	SAL	LPS	SAL	LPS	SAL		*P*_DAR_	*P*_stress_	*P*_D*S_		
Ca	886.14	548.05	757.36	444.94	501.06	808.66	676.50	433.16	523.10	438.81	38.16	0.379	0.095	0.088	NS	NS
S	7,964	5,326	7,390	3,577	5,095	5,229	5,351	5,235	7,050	5,380	368.17	0.684	0.038	0.434	NS	NS
Mg	169.61	128.46	176.96	108.27	145.03	165.15	151.72	95.68	118.25	99.10	6.99	0.249	0.027	0.357	−0.131	−0.090
Cu	18.11^C^	7.47	9.10^C^	11.66	7.41^C^	8.13	6.86^B^	44.77	58.99^A^	47.34	1.67	<0.0001	0.279	0.000	<0.0001	<0.0001
Fe	173.35^B^	126.28	98.46^B^	60.56	85.90^B^	125.01	261.27^A^	12,102	8,588^A^	14,655	863.02	0.000	0.052	0.147	0.000	<0.0001
Mn	6.59^B^	1.29	1.66^B^	1.22	1.35^B^	1.95	5.98^B^	43.89	31.97^A^	86.69	5.09	0.003	0.102	0.270	0.000	<0.0001
Zn	147.72^BC^	57.51	81.37^C^	79.64	63.50^C^	79.85	117.30^B^	174.49	257.85^A^	241.75	7.84	<0.0001	0.672	0.072	<0.0001	<0.0001

1 LPS, lipopolysaccharide, SAL, saline, SEM, standard error of the mean, DAR, dietary amylose/amylopectin ratio. L, Q represents a linear, quadratic response to increasing dietary amylose/amylopectin ratio. Only data from non-stress piglets were analyzed in this REG procedure.

^A-CValues within a row with different superscripts differ significantly at p < 0.05. NS means a p-value of > 0.15.^

### 3.8 Indole and skatole contents in chyme

Cecal indole concentration in the DAR 0.00 group was higher than that in the DAR 0.80 group (*p* < 0.05). It was not affected by LPS stress (*p* > 0.05) (Table 8). Cecal skatole and colonic indole were not affected by DAR or LPS stress (*p* > 0.05). Piglets that had DAR 0.00 had lower colonic skatole than those from the DAR 0.40, 0.60, and 0.80 groups (*p* < 0.05). Colonic skatole concentration increased after LPS stress in DAR 0.40 and 0.80 but decreased in other groups (*p* < 0.05). No variables were selected in the REG procedure at a *p*-value of <0.15.

**Table 8:** Effects of dietary amylose/amylopectin ratio on indole and skatole concentrations in cecal and colonic chyme of weaned piglets under LPS stress.^1^

Items	0.00		0.20		0.40		0.60		*0.80*		*SEM*	*p* value			*P_L_*	*P_Q_*
	LPS^1^	SAL	LPS	SAL	LPS	SAL	LPS	SAL	LPS	SAL		*P*_DAR_	*P*_stress_	*P*_D*S_	SAL	
Indol(e), μg/g																
Cecal chyme	11.79^A^	9.71	19.24^AB^	3.62	9.84^AB^	6.54	3.47^AB^	2.86	0.00^B^	4.07	1.01	0.031	0.123	0.091	NS	NS
Colonic chyme	2.73	3.21	5.47	14.37	10.05	7.43	4.00	8.97	5.28	4.90	0.94	0.246	0.271	0.402	NS	NS
Skatole, μg/g																
Cecal chyme	11.01	8.93	23.64	13.57	20.69	6.59	14.17	5.44	0.35	8.78	1.46	0.102	0.108	0.214	NS	NS
Colonic chyme	3.59^C^	8.18	8.81^BC^	28.18	40.14^AB^	23.62	30.56^A^	44.94	39.81^AB^	15.88	2.20	0.002	0.930	0.019	NS	NS

1 LPS, lipopolysaccharide, SAL, saline, SEM, standard error of the mean, DAR, dietary amylose/amylopectin ratio. L, Q represents a linear, quadratic response to increasing dietary amylose/amylopectin ratio. Only data from non-stress piglets were analyzed in this REG procedure.

^A-CValues within a row with different superscripts differ significantly at p < 0.05. NS means a p-value of > 0.15.^

## 4 Discussion

When weaned piglets experienced LPS stress, serum AST activity and urea nitrogen levels increased, while serum glucose and glutamate concentrations decreased. In contrast, hepatic concentrations of Cys, Glu, and Gly increased. Serum AST activity is a key biochemical marker of liver health and function. The observed elevation of serum AST levels following LPS stress (20) indicates hepatic injury occurred under these situations.

In response to LPS stress, piglets may redirect AA from protein retention toward AA utilization in the immune response. The increase in serum urea nitrogen in this study can be associated with elevated AA catabolism, particularly in amino acids in excess. Specific AAs are utilized in the synthesis of immune system metabolites, such as immunoglobulins and glutathione, during LPS stress (6). This was confirmed by the elevated GSH-PX activity, upregulated *GPX1* gene expression in the liver, and increased plasma Cys flux in pigs challenged with LPS in this study.

Compared to the diet 0.00, piglets consuming a diet high in amylose (0.80) exhibited higher serum Arg, Cit, and glucose concentrations. Both hepatic T-SOD activity and SOD1 mRNA expression were also elevated in the 0.80 group after LPS stress. Additionally, serum IgG levels increased in piglets on a diet of 0.80 after LPS stress. Copper, zinc-superoxide dismutase (SOD1), a major intracellular antioxidant enzyme in mammals, plays a crucial role in mitigating LPS-induced hepatic protein nitration (21). The fermentation of raw potato starch (RS2) in the cecum enhances the intestinal absorption of minerals such as Ca, Mg, Fe, Zn, and Cu (22). Given the higher amylose content in diet 0.80, piglets from this group had significantly higher hepatic Cu concentrations after LPS stress compared to other groups. While Prates et al. (7) reported that plasma IgG and IgM concentrations in weaned piglets were reduced 3 days after an LPS challenge, the increased IgG observed in the 0.80 group in this study suggests enhanced protection against oxidative stress and inflammation. Indoxyl sulfate and p-cresol sulfate, major microbial-derived pro-inflammatory and pro-oxidant uremic toxins, decrease under lower pH conditions (23). The lower pH associated with the diet 0.80 (24) likely contributed to the observed decrease in cecal indoles in this study. However, colonic skatole concentrations increased in the diet 0.80 group after LPS stress. A diet high in amylose may lead to the reorganization of the intestinal mucosa, thereby increasing gut cell debris availability for microbial skatole production from tryptophan (25). We speculate that piglets consuming the diet 0.80 were better protected against LPS stress due to enhanced antioxidant capacity and immune response.

Although average feed intake was the same across all groups (24), serum-free AAs such as Arg, Ala, Asp, Pro, Cit, Cysthi, a-ABA, and EOHNH2 were either higher or at least not lower in DAR 0.20 compared to other groups at D15. However, by day 29, only serum Pro, Sar, Cit, and Hypro concentrations remained elevated in DAR 0.20 relative to the other groups.

After 24 h of incubation with cecal and colonic digesta, the production of ammonia-nitrogen and branched-chain fatty acids (BCFAs), markers of protein fermentation, decreased as corn-resistant starch levels increased (26). In the DAR 0.20 group, protein fermentation in the large intestine was evident after feed transition, as indicated by significant increases in isobutyrate and isovalerate concentrations (24). Protein fermentation products have been associated with toxic and pro-inflammatory effects on the intestinal epithelium (27), potentially explaining the highest hepatic MDA concentration observed in the diet 0.20. Normally, liver protein synthesis increases when animals undergo immune challenges. However, the higher protein fermentation in DAR 0.20 likely contributed to reductions in hepatic Ile and Leu levels after LPS stress, as BCFAs such as iso-butyrate, 2-methyl-butyrate, and iso-valerate are products of valine, isoleucine, and leucine deamination. Notably, serum Ser concentrations decreased after LPS stress in the DAR 0.20 and 0.80 groups while increasing in the other groups. Serine plays a critical role in metabolic networks interlinking the folate and methionine cycles, supporting cell proliferation (28).

It also serves as a precursor for Gly and Cys, which contribute to the synthesis of the antioxidant glutathione (GSH). Conversely, hepatic amino acids such as Phe, Thr, Asp, Ser, Glu, and Gly were likely mobilized to counteract oxidative stress, as indicated by elevated hepatic MDA levels. Increasing the availability of amino acids entering the portal vein has been suggested to enhance tissue protein synthesis (29). Higher hepatic Cys levels in the DAR 0.20 group suggest active Glu synthesis in this group. Additionally, DAR 0.20 exhibited the highest GSH-PX activity in the liver among all groups, further underscoring its role in combating oxidative stress.

## 5 Conclusion

In conclusion, feeding weaned piglets a diet high in amylose (DAR 0.80) enhances their ability to cope with LPS stress by mobilizing amino acids for IgG synthesis and improving antioxidant function. This finding provides a new strategy to protect piglets from LPS-induced stress by regulating dietary starch structure.

## Data Availability

The raw data supporting the conclusions of this article will be made available by the authors without undue reservation.
