# Supplementary material for: Effects of dietary amylose/amylopectin ratio on antioxidant ability and amino metabolism in the liver of weaned piglets undergoing feed transition and challenged with lipopolysaccharide
Source: Front Nutr. 2025 Jan 6;11:1435051. doi: 10.3389/fnut.2024.1435051 (PMC11743269; doi:10.3389/fnut.2024.1435051)
Supplement: Supplementary file 1 [file Table_1.docx]

**Table S1 Composition of experimental diets (as fed basis)**

| Ingredients, % | Pre-care period (7～11 kg) | | | | | Late-care period (11～25 kg) | | | | |
| --- | --- | --- | --- | --- | --- | --- | --- | --- | --- | --- |
|  | 0.00 | 0.20 | 0.40 | 0.60 | 0.80 | 0.00 | 0.20 | 0.40 | 0.60 | 0.80 |
| Waxy corn starch | 54.80 | 45.21 | 38.36 | 33.43 | 29.32 | 53.54 | 44.17 | 37.48 | 32.66 | 28.64 |
| High-Maize 1043 | - | 9.59 | 16.44 | 21.37 | 25.48 | - | 9.37 | 16.06 | 20.88 | 24.90 |
| Soybean meal | 9.00 | 9.00 | 9.00 | 9.00 | 9.00 | 12.00 | 12.00 | 12.00 | 12.00 | 12.00 |
| Full fat expanded soybean meal | 9.00 | 9.00 | 9.00 | 9.00 | 9.00 | 10.00 | 10.00 | 10.00 | 10.00 | 10.00 |
| Fermented soybean meal | 8.00 | 8.00 | 8.00 | 8.00 | 8.00 | 10.00 | 10.00 | 10.00 | 10.00 | 10.00 |
| Fish meal | 5.00 | 5.00 | 5.00 | 5.00 | 5.00 | 5.80 | 5.80 | 5.80 | 5.80 | 5.80 |
| Whey, dried | 5.00 | 5.00 | 5.00 | 5.00 | 5.00 | 5.00 | 5.00 | 5.00 | 5.00 | 5.00 |
| Plasma protein powder | 4.84 | 4.84 | 4.84 | 4.84 | 4.84 | - | - | - | - | - |
| Monocalcium Phosphate | 1.17 | 1.17 | 1.17 | 1.17 | 1.17 | 1.33 | 1.33 | 1.33 | 1.33 | 1.33 |
| Soybean oil | 1.00 | 1.00 | 1.00 | 1.00 | 1.00 | - | - | - | - | - |
| Premix^1^ | 0.92 | 0.92 | 0.92 | 0.92 | 0.92 | 0.92 | 0.92 | 0.92 | 0.92 | 0.92 |
| Choline chloride | 0.08 | 0.08 | 0.08 | 0.08 | 0.08 | 0.08 | 0.08 | 0.08 | 0.08 | 0.08 |
| Limestone | 0.74 | 0.74 | 0.74 | 0.74 | 0.74 | 0.72 | 0.72 | 0.72 | 0.72 | 0.72 |
| DL-lysine | 0.23 | 0.23 | 0.23 | 0.23 | 0.23 | 0.21 | 0.21 | 0.21 | 0.21 | 0.21 |
| DL Methionine | 0.22 | 0.22 | 0.22 | 0.22 | 0.22 | 0.21 | 0.21 | 0.21 | 0.21 | 0.21 |
| Salt | - | - | - | - | - | 0.19 | 0.19 | 0.19 | 0.19 | 0.19 |
| TOTAL | 100 | 100 | 100 | 100 | 100 | 100 | 100 | 100 | 100 | 100 |
| Calculated nutrient content^2^ |  |  |  |  |  |  |  |  |  |  |
| Amylose/amylopectin ratio | 0.00 | 0.20 | 0.40 | 0.60 | 0.80 | 0.00 | 0.20 | 0.40 | 0.60 | 0.80 |
| Digestive energy, kcal/kg | 3500 | 3500 | 3500 | 3500 | 3500 | 3408 | 3408 | 3408 | 3408 | 3408 |
| Crude protein,% | 18.50 | 18.50 | 18.50 | 18.50 | 18.50 | 18.00 | 18.00 | 18.00 | 18.00 | 18.00 |
| Ca,% | 0.85 | 0.85 | 0.85 | 0.85 | 0.85 | 0.92 | 0.92 | 0.92 | 0.92 | 0.92 |
| Av.P,% | 0.42 | 0.42 | 0.42 | 0.42 | 0.42 | 0.48 | 0.48 | 0.48 | 0.48 | 0.48 |
| Salt,% | 0.50 | 0.50 | 0.50 | 0.50 | 0.50 | 0.50 | 0.50 | 0.50 | 0.50 | 0.50 |
| Amino acids, %SID^3^ |  |  |  |  |  |  |  |  |  |  |
| Lys | 1.45 | 1.45 | 1.45 | 1.45 | 1.45 | 1.31 | 1.31 | 1.31 | 1.31 | 1.31 |
| TSAA | 0.79 | 0.79 | 0.79 | 0.79 | 0.79 | 0.71 | 0.71 | 0.71 | 0.71 | 0.71 |
| Thr | 0.83 | 0.83 | 0.83 | 0.83 | 0.83 | 0.74 | 0.74 | 0.74 | 0.74 | 0.74 |
| Trp | 0.25 | 0.25 | 0.25 | 0.25 | 0.25 | 0.23 | 0.23 | 0.23 | 0.23 | 0.23 |

^1^Supplied per kg of complete diet: 2200 IU vitamin A,16 mg vitamin E, 220 IU vitamin D, 0.5 mg vitamin K,1.0 mg thiamin,3.5 mg riboflavin,30 mg niacin, 7 mg pyridoxine, 10 mg d-pantothenic acid, and 17.5 μg vitamin B_12_. Fe as FeSO_4_·7H_2_O,100 mg; Mn as MnSO_4_·7H_2_O, 4 mg; Zn as ZnSO_4_·7H_2_O, 100 mg; Cu as CuSO_4_·5H_2_O, 6 mg; Se as NaSeO_3_, 0.3 mg; and I as KI,0.14 mg.

^2^Nutrient content of diets based on estimted nutrient contents of ingredients according to NRC(2012).

^3^SID, Standardized ileal digestible.Dietary amylose/amylopectin ratio of A, B, C, D, E was 0.00, 0.20, 0.40,0.60 and 0.80 respectively.

**Table S2 Primers used for real-time PCR analysis**

| Genes^1^ | Primers | Sequences(5'-3') | Size, bp | GeneBank accession No. |
| --- | --- | --- | --- | --- |
| *β*-*actin* | Forward | AGTTGAAGGTGGTCTCGTGG | 216 | XM_003357928.4 |
|  | Reverse | TGCGGGACATCAAGGAGAAG | | |
| *GCLC* | Forward | CAAACCATCCTACCCTTTGG | 172 | XM_021098556.1 |
|  | Reverse | ATTGTGCAGAGAGCCTGGTT | | |
| *Nrf2* | Forward | GAAAGCCCAGTCTTCATTGC | 190 | XM_021075133.1 |
|  | Reverse | TTGGAACCGTGCTAGTCTCA | | |
| *SOD1* | Forward | GAGACCTGGGCAATGTGACT | 189 | NM_001190422.1 |
|  | Reverse | CCAAACGACTTCCAGCATTT | | |
| *GPX1* | Forward | AGCCCAACTTCATGCTCTTC | 159 | NM_214201.1 |
|  | Reverse | CATTGCGACACACTGGAGAC | | |

*^1^GCLC:* glutamate-cysteine ligase catalytic subunit; *Nrf2:* nuclear factor, erythroid 2 like 2; *SOD1:* superoxide dismutase; *GPX1:* and glutathione peroxidase 1.
